# The Ciidae (Coleoptera) of New Brunswick, Canada: New records and new synonyms

**DOI:** 10.3897/zookeys.573.7445

**Published:** 2016-03-24

**Authors:** Cristiano Lopes-Andrade, Reginald P. Webster, Vincent L. Webster, Chantelle A. Alderson, Cory C. Hughes, Jon D. Sweeney

**Affiliations:** 1 Laboratório de Sistemática e Biologia de Coleoptera, Departamento de Biologia Animal, Universidade Federal de Viçosa, 36570-900, Viçosa, MG, Brasil; 2 24 Mill Stream Drive, Charters Settlement, NB, Canada E3C; 3 Natural Resources Canada, Canadian Forest Service - Atlantic Forestry Centre, 1350 Regent St., P.O. Box 4000, Fredericton, NB, Canada E3B 5P7

**Keywords:** Ciidae, new synonyms, new records, Canada, New Brunswick

## Abstract

The Ciidae of New Brunswick, Canada are reviewed. Seventeen species are recorded for New Brunswick, including the following 10 species that are newly recorded for the province: *Ceracis singularis* (Dury), *Ceracis thoracicornis* (Ziegler), *Cis angustus* Hatch, *Cis fuscipes* Mellié, *Cis horridulus* Casey, *Cis striatulus* Mellié, *Dolichocis laricinus* (Mellié), *Malacocis brevicollis* (Casey), *Orthocis punctatus* (Mellié), and *Plesiocis cribrum* Casey. Additional locality data are provided for the following species previously known from the province: *Cis americanus* Mannerheim, *Cis creberrimus* Mellié, *Cis levettei* (Casey), *Cis submicans* Abeille de Perrin, *Dolichocis manitoba* Dury, *Hadreule elongatula* (Gyllenhal), and *Octotemnus glabriculus* (Gyllenhal). Seven synonyms are proposed here; *Cis pistoria* Casey with *Cis submicans* Abeille de Perrin; *Cis fraternus* Casey, *Cis macilentus* Casey and *Cis striolatus* Casey with *Cis striatulus* Mellié; *Dolichocis indistinctus* Hatch with *Dolichocis laricinus* (Mellié); and *Octotemnus denudatus* Casey and *Octotemnus laevis* Casey with *Octotemnus glabriculus* (Gyllenhal). Lindgren funnel traps provided the majority of specimens for 15 of the 17 species reported from New Brunswick and were the sole source of specimens for seven of the 10 species newly reported here, suggesting they are a very useful tool for sampling Ciidae in the forests of New Brunswick.

## Introduction

The systematics, taxonomy, and biology of the North American Ciidae (minute tree-fungus beetles) are well known as a result of the works by Lawrence (1965, 1967, 1971, 1973, 1982) and Thayer and Lawrence (2002). Adults and larvae live in and feed on basidiomes of various species of basidiomycete fungi, commonly known as polypores or bracket fungi, with most ciid species limited to a few host species (Lawrence 1973, Thayer and Lawrence 2002, Orledge and Reynolds 2005).

The Ciidae of the Maritime Provinces of Canada were reviewed by Majka (2007). He provided an overview of the history of collecting, host usage of species occurring in the region, and a discussion on the distribution and zoogeography of the fauna. Fifteen species were reported from the region, including six species from New Brunswick. *Cis pistoria* Casey was newly recorded from New Brunswick. Majka (2007) noted that sampling in New Brunswick was inadequate and that additional species would likely be documented with more intensive sampling.

During a study in New Brunswick to develop tools for improved detection of invasive species of Cerambycidae, many Ciidae were collected from Lindgren funnel trap samples. Other specimens were collected during general sampling, and additional material was found in several museum collections. Ten species new to New Brunswick were found during this survey as well as many additional records of species previously known from the province. The purpose of this paper is to document these records and to present seven new synonyms.

## Methods and conventions

**Collection methods.** The following records are based, in part, on specimens collected as part of a general survey to document the Coleopteran fauna of New Brunswick. Other records were obtained from specimens contained in the collections listed below. Most species records came from samples collected from Lindgren funnel traps deployed at 27 sites (24–64 traps per site) between 2009 and 2015. At many sites, starting during 2012, traps were deployed in the upper canopy as well as in the understory, usually in equal numbers, although at a few sites, only canopy traps or understory traps were used. Canopy traps were 10–20 m above the ground, whereas understory traps were 1–1.5 m above the ground (i.e., 30–50 cm from the bottom of the collecting cup to the ground). In both cases, traps were suspended from a rope such that the trap was at least 1 m from the main stem of trees and at least 30 m from another trap. Traps were baited with various combinations of lures for detecting Cerambycidae. However, data on attractants were not collected for the Ciidae. For details of the methods used to deploy Lindgren traps, for sample collection, and lure combinations used, see Webster et al. (2012a, 2012b), Hughes et al. (2014), and Webster et al. (2016). Locality and habitat data are presented as on labels for each record. Two labels were used on many specimens, one that included the locality, collection date, and collector, and one with macro- and microhabitat data and collection method. Information from the two labels is separated by a // in the data presented from each specimen.

**Distribution.** Every species is cited with current distribution in Canada and Alaska, using abbreviations for the state, provinces, and territories. New records for New Brunswick are indicated in **bold** under **Distribution in Canada and Alaska**. The following abbreviations are used in the text:

AB Alberta


AK Alaska


BC British Columbia


MB Manitoba


NB New Brunswick


NF & LB Newfoundland and Labrador*


NS Nova Scotia


NT Northwest Territories


NU Nunavut


ON Ontario


PE Prince Edward Island


QC Quebec


SK Saskatchewan


YT Yukon Territory


*Newfoundland and Labrador are each treated separately under the current Distribution in Canada and Alaska.

**Taxonomy.** Specimens were initially separated into morphospecies and then identified using keys to North American Ciidae provided by Lawrence (1967, 1971). They were also carefully compared with identified specimens deposited in the Coleção Entomológica do Laboratório de Sistemática e Biologia de Coleoptera, Universidade Federal de Viçosa, Viçosa, Minas Gerais, Brasil (CELC) and with material borrowed from North American and European museums, which included material compared with types, collected in type localities, and identified by former and current specialists on Nearctic and Palaearctic ciids (e.g., Adolf Lohse, Alexander Kompantsev, Glenda Orledge, Johannes Reibnitz, John Lawrence, Makoto Kawanabe, among others). As the North American Ciidae fauna were completely and carefully revised by Lawrence (1971), who examined most types, proposed synonyms, and redelimited species, identification of North American species is reliable, and the morphological limits of most species are well understood. The major problems that persisted in the taxonomy of North American ciids are also mentioned and discussed in that work, mainly regarding a few possible cases of conspecificity between North American and European species. In order to check these, the ciid species from NB were compared with Holarctic material and data in literature (e.g. Lohse 1967, Kawanabe 2002). When necessary and possible, males were dissected, and their genitalia mounted on slides following the protocol provided by Lopes-Andrade (2011). A complete list of references and original combinations is provided only for species with new synonyms proposed here.

It is important to emphasize that we propose new synonyms only for species with well-known morphological limits and that were previously studied by authors who conducted faunistic or revisionary works on the North American or the European Ciidae faunas. The morphological limits of these species are well established in literature, there is available material deposited in museums and used for comparison, and there remains no doubt on their identification. For instance, there is no doubt that North American specimens currently identified as *Octotemnus laevis* all refer to a single species, and its definition was revised previously by Lawrence (1971). There is no doubt that European and Asian ciids identified as *Octotemnus glabriculus* all refer to a single species, and the unique species that could be confounded with it, *Octotemnus rugosopunctatus* Drogvalenko, was also examined. But there is robust evidence that *Octotemnus laevis* and *Octotemnus glabriculus* refer to a single, Holarctic species, and thus it was necessary to resolve this so as not to perpetuate the problem. All other synonyms proposed here follow this same reasoning. In the case of *Orthocis punctatus*, a cryptic species for which we do not have enough material identified by renowned specialists or compared with type specimens, we have retained the name used by Lawrence (1971). However, it is likely that further synonyms will arise as relevant material is examined.

Some species names cited here were proposed by Mellié in a work published in two separate parts, and in the last decades, there has been much confusion in the literature regarding the publication year of his monograph. Authors have cited both parts of Mellié’s monograph as being published in either 1848 or 1849, or 1848 for the first part and 1849 for the second. Here, we used 1849 as the publication date for both parts of Mellié’s work, following Orledge and Booth (2006) who stated “(...) it is clear that pages 313–396 [part 2] were published in April/May 1849, and that pages 205–274 [part 1] may well have been published in January 1849.” A more detailed discussion on this matter can be found in Orledge and Booth (2006), but it is important to emphasize here that subsequent authors are following their suggestion (e.g., Jelínek 2007, Reibnitz et al. 2013, Sandoval-Gómez et al. 2014).

**Acronyms of collections referred to in this study are as follows**:

AFC
 Atlantic Forestry Centre, Fredericton, New Brunswick, Canada 


CELC
 Coleção Entomológica do Laboratório de Sistemática e Biologia de Coleoptera, Universidade Federal de Viçosa, Viçosa, Minas Gerais, Brasil 


CNC
 Canadian National Collection of Insects, Arachnids and Nematodes, Ottawa, Ontario, Canada 


NBM
 New Brunswick Museum, Saint John, New Brunswick, Canada 


RWC
 Reginald P. Webster Collection, Charters Settlement, New Brunswick, Canada 


UMC
University of Moncton, Moncton, New Brunswick, Canada 


**Photography.** Individuals were photographed under a Zeiss V8 stereomicroscope equipped with a Zeiss AxioCam MRc (Figs 1–4, 7, 10, 15) or under a Zeiss V20 equipped with a Zeiss AxioCam 506 (Figs 5–6, 8–9, 11–14, 16). All studied species from NB were photographed, except for *Cis creberrimus* Mellié, which we were unable to examine.

**Figures 1–5.**
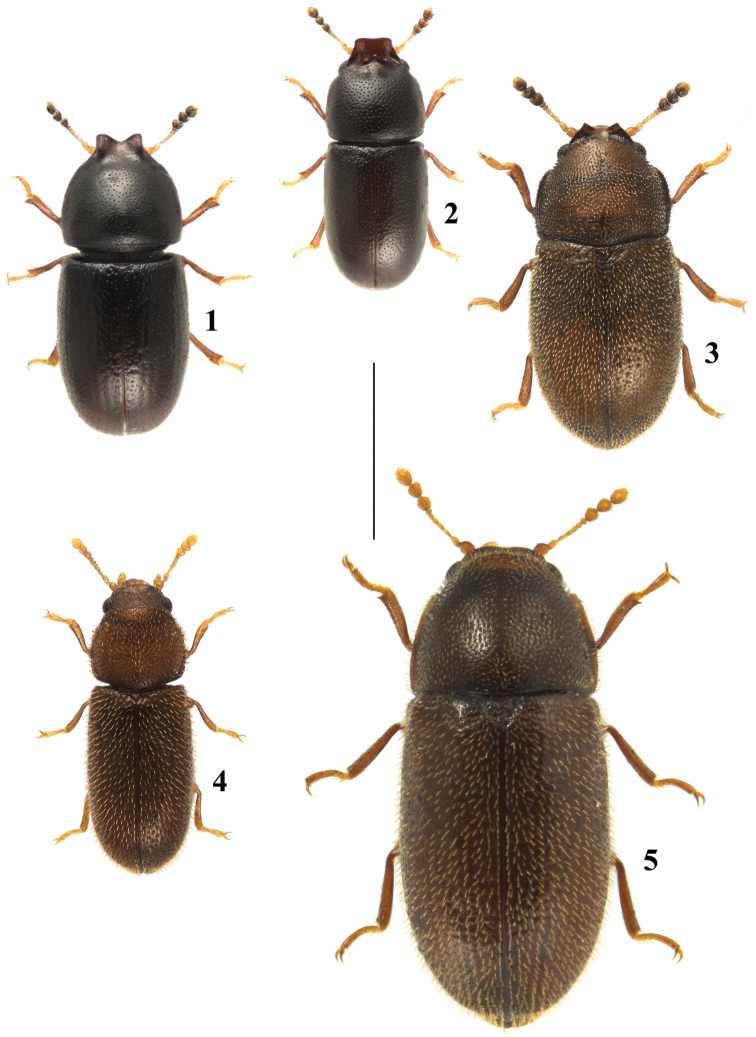
Dorsal view of species from New Brunswick, Canada. **1** *Ceracis singularis* (Dury) **2** *Ceracis thoracicornis* (Ziegler) **3** *Cis americanus* Mannerheim **4** *Cis angustus* Hatch **5** *Cis fuscipes* Mellié. Scale bar: 1 mm.

**Figures 6–9.**
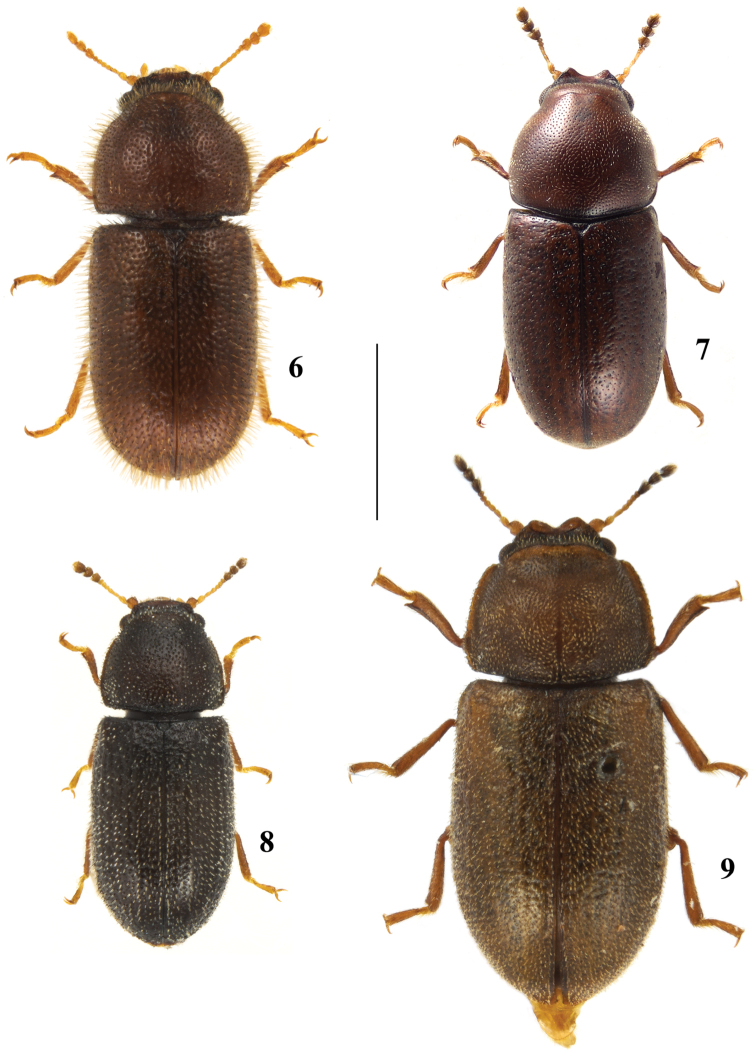
Dorsal view of species from New Brunswick, Canada. **6** *Cis horridulus* Casey **7** *Cis levettei* (Casey) **8** *Cis striatulus* Mellié **9** *Cis submicans* Abeille de Perrin. Scale bar: 1 mm.

**Figures 10–16.**
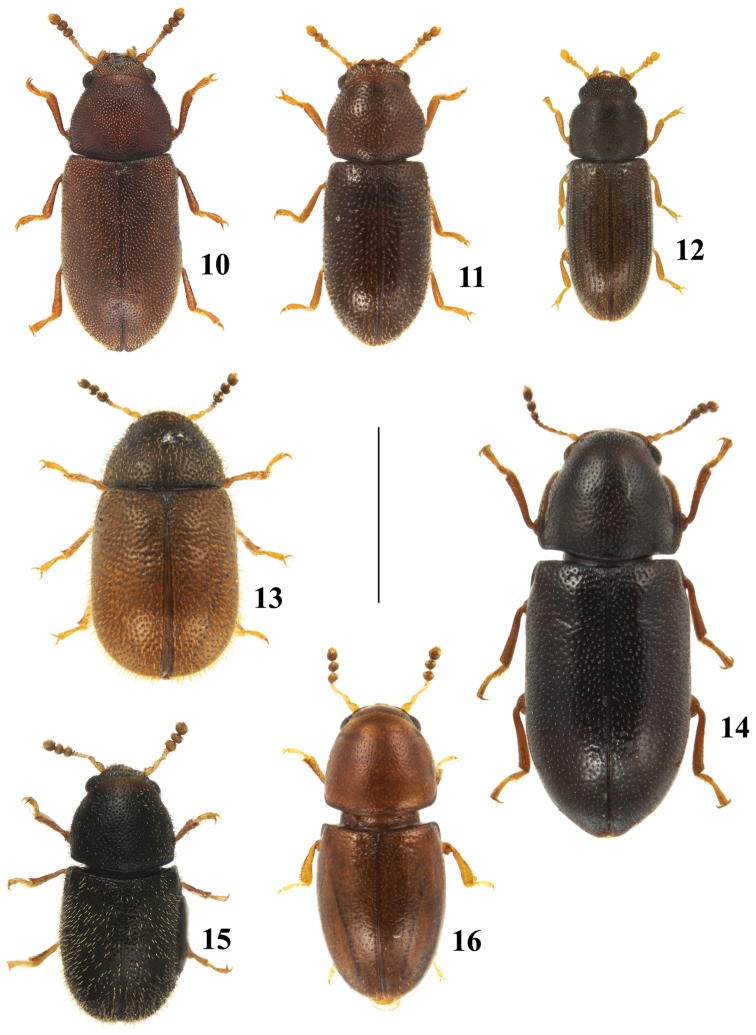
Dorsal view of species from New Brunswick, Canada. **10** *Dolichocis laricinus* (Mellié) **11** *Dolichocis manitoba* Dury **12** *Hadreule elongatula* (Gyllenhal) **13** *Malacocis brevicollis* (Casey) **14** *Orthocis punctatus* (Mellié) **15** *Plesiocis cribrum* Casey **16** *Octotemnus glabriculus* (Gyllenhal). Scale bar: 1 mm.

## Results

### Species accounts

Species with a † are adventive to Canada, species with a * are Holarctic. The determination that a species was a new record was based on information in the print version of Bousquet et al. (2013). Four of the species found in NB are new junior synonyms of four Palaearctic names, respectively: *Cis pistoria* Casey with *Cis submicans* Abeille de Perrin; *Cis striolatus* Casey with *Cis striatulus* Mellié; *Dolichocis indistinctus* Hatch with *Dolichocis laricinus* (Mellié); and *Octotemnus laevis* Casey with *Octotemnus glabriculus* (Gyllenhal). However, in two cases, the junior synonyms proposed here already had previously proposed synonyms to them: *Cis fraternus* Casey and *Cis macilentus* Casey, previously synonymized to *Cis striolata*; *Octotemnus denudatus* Casey, previously synonymized to *Octotemnus laevis*. These were well-established synonyms, and we also include these names as new synonyms of *Cis striatulus* and *Octotemnus glabriculus*, respectively. We follow recent alterations on a few names of European species provided by Jelínek (2007).

### Family Ciidae Leach, 1819

#### Subfamily Ciinae Leach, 1819

##### Tribe Ciini Leach, 1819

###### Ceracis singularis

Taxon classificationAnimaliaColeopteraCiidae

(Dury, 1917) new to New Brunswick

Fig. 1

####### Material examined.

**New Brunswick, Gloucester Co.**, Bathurst, Daly Point Nature Preserve, 47.6392°N, 65.6098°W, 15–25.VI.2015, 9–23.VII.2015, C. Alderson & V. Webster // Mixed forest, green Lindgren funnel trap 1 m high (1), black Lindgren funnel trap 1 m high (1) (2, RWC). **Kent Co.**, Kouchibouguac National Park, 46.8087°N, 64.9078°W, 24.VI-7.VII.2015, 7–22.VII.2015, C. Alderson & V. Webster // Poplar/red maple stand, Lindgren funnel trap, 1 m high (1, AFC; 1, RWC). **Queens Co.**, Cranberry Lake P.N.A. [Protected Natural Area], 46.1125°N, 65.6075°W, 12–29.VI.2012, R. Webster & M.-A. Giguère // Red oak forest, Lindgren funnel trap (1, RWC). **Sunbury Co.**, Gilbert Island, 45.8770°N, 66.2954°W, 12–29.VI.2013, C. Alderson, C. Hughes, & V. Webster // Hardwood forest, Lindgren funnel trap 1 m high under *Tilia americana* (1, RWC); Sunpoke Lake, 45.7656°N, 66.5550°W, 18.VI-9.VII.2012, C. Alderson & V. Webster // Red oak forest near seasonally flooded marsh, Lindgren funnel trap 1 m high under *Quercus rubra* (1, RWC). **York Co.**, 15 km W of Tracy off Rt. 645, 45.6848°N, 66.8821°W, 20–29.VII.2009, R. Webster & M.-A. Giguère // Red pine forest, Lindgren funnel trap (1, RWC); same locality data and forest type but 20.VI-6.VII.2011, M. Roy & V. Webster // Flight intercept trap (1, RWC); Keswick Ridge, 45.9962°N, 66.8781°W, 19.VI-3.VII.2014, C. Alderson & V. Webster // Mixed forest, Lindgren funnel trap 1 m high under trees (1, RWC)

####### Distribution in Canada and Alaska.

ON, QC, **NB** (Bousquet et al. 2013). All new records of *Ceracis singularis* (Dury) in NB were based on specimens captured in Lindgren funnel traps or a flight intercept trap. This species is widespread in the province (seven localities) but was captured in low numbers at each site. This species was found in hardwood, mixed, and pine forests. These are the first records of this species from the Maritime Provinces of Canada.

###### Ceracis thoracicornis

Taxon classificationAnimaliaColeopteraCiidae

(Ziegler, 1845) new to New Brunswick

Fig. 2

####### Material examined.

**New Brunswick, Carleton Co.**, Jackson Falls, “Bell Forest”, 46.2200°N, 67.7231°W, 16–21.VI.2009, R. Webster & M.-A. Giguère // Rich Appalachian hardwood forest with some conifers, Lindgren funnel trap (1, RWC). **Kent Co.**, Kouchibouguac National Park, 46.8072°N, 64.9100°W, 24.VI-7.VII.2015, C. Alderson & V. Webster // Jackpine forest, Lindgren funnel traps, 1 m high (2, RWC). **Queens Co.**, C.F.B. Gagetown, 45.7516°N, 66.1866°W, 17.VI-3.VII.2013, 30.VII-14.VIII.2015, C. Alderson & V. Webster // Old mixed forest with *Quercus rubra*, Lindgren funnel traps in canopy of *Quercus rubra* (3), in canopy (1) (4, RWC). **York Co.**, Keswick Ridge, 45.9962°N, 66.8781°W, 19.VI-3.VII.2014, C. Alderson & V. Webster // Mixed forest, Lindgren funnel trap 1 m high under trees (1, RWC); 16 km W of Tracy off Rt. 645, 45.6854°N, 66.8839°W, 11–25.VII.2014, C. Alderson & V. Webster // Old red pine forest, Lindgren funnel trap (1, RWC).

####### Distribution in Canada and Alaska.

MB, ON, QC, **NB**, NS (Bousquet et al. 2013). All new records of *Ceracis thoracicornis* (Ziegler) in NB were based on specimens captured in Lindgren funnel traps. This species is widespread in the southern half of the province but was found at only five localities and in relatively low numbers. This species was found in hardwood, mixed, and pine forests.

###### Cis americanus

Taxon classificationAnimaliaColeopteraCiidae

Mannerheim, 1852

Fig. 3

####### Material examined.

**New Brunswick, Kent Co.**, Kouchibouguac National Park, 46.8072°N, 64.9100°W, 21–27.V.2015, C. Alderson & V. Webster // Jackpine forest, Lindgren funnel trap, 1 m high (1, AFC); same locality and collectors but 46.8087°N, 64.9078°W, 27.V-12.VI.2015, C. Alderson & V. Webster // Poplar/red maple stand, Lindgren funnel trap, 1 m high (1, RWC). **Northumberland Co.**, Ludlow, 14.VI.1967, D. P. Pielou, Ex: *Polyporus betulinus*, A-74 (1, CNC); ca. 2.5 km W of Sevogle, 47.0876°N, 65.8613°W, 11–26.VI.2013, C. Alderson & V. Webster // Old *Pinus banksiana* stand, Lindgren funnel trap (1, RWC). **Queens Co.**, Cranberry Lake P.N.A., 46.1125°N, 65.6075°W, 25.VI-1.VII.2009, R. Webster & M.-A. Giguère // Red oak forest, Lindgren funnel trap (1, RWC); same locality data and forest type but 25.V-7.VI.2011, 29.VI-7.VII.2011, M. Roy & V. Webster // Lindgren funnel traps (2, RWC); same locality data, forest type and trap but 7–22.VI.2011 (1, CELC). **Restigouche Co.**, Dionne Brook P.N.A., 47.9030°N, 68.3503°W, 30.V-15.VI.2011, M. Roy & V. Webster // Old-growth northern hardwood forest, Lindgren funnel trap (1, RWC). **Sunbury Co.**, Wirral, 16.V.1967 (H413), 11.VII.1967 (H417 & H483), 18.VIII.1967 (H407), 1.IX.1967 (H414), 15.X.1968 (H773), D.P. Pielou, Ex: *Polyporus betulinus* (6, CNC); Acadia Research Forest, 45.9866°N, 66.3441°W, 19–25.V.2009, 16–24.VI.2009, R.P. Webster & M.-A. Giguère // Red spruce forest with red maple & balsam fir, Lindgren funnel traps (1, AFC; 1, RWC); same locality but 45.9912°N, 66.2668°W, 15–25.VI.2012 // Mature mixed forest with balsam fir, red maple & scattered white pine, tamarack & large tooth aspen, Lindgren funnel trap (1, RWC). **York Co.**, 15 km W of Tracy off Rt. 645, 45.6848°N, 66.8821°W, 13–19.V.2009, 20–29.VII.2009, R. Webster & M.-A. Giguère // Old red pine forest, Lindgren funnel traps (1, AFC; 1, RWC); 14 km WSW of Tracy S of Rt. 645, 45.6741°N, 66.8661°W, 10–26.V.2010, R. Webster & C. MacKay, coll. // Old mixed forest with red & white spruce, red & white pine, balsam fir, eastern white cedar, red maple & *Populus* sp., Lindgren funnel traps (2, RWC); Fredericton, Odell Park, 45.9484°N, 66.6802°W, 22.V-4.VI.2014, C. Alderson & V. Webster // Old mixed forest, Lindgren funnel trap in front of tree hole (1, NBM).

####### Distribution in Canada and Alaska.

AK, BC, AB, SK, MB, ON, QC, NB, NS, PE, NF (Bousquet et al. 2013). Nearly all records of *Cis americanus* Mannerheim, which is widespread (12 sites) in NB, were based on specimens captured in Lindgren funnel traps from various forest types. The first records of this species from NB were specimens reared from *Piptoporus betulinus* (Bull.) Fr. (=*Polyporus betulinus*) (birch polypore) (Polyporaceae) from two localities (Wirral and Ludlow) (Pielou and Verna 1968).

###### Cis angustus

Taxon classificationAnimaliaColeopteraCiidae

Hatch, 1962 new to New Brunswick

Fig. 4

####### Material examined.

**New Brunswick, Carleton Co.**, Meduxnekeaq Valley Nature Preserve. 46.1907°N, 67.6740°W, 3–17.VII.2012, C. Alderson & V. Webster // Old mixed forest, Lindgren funnel trap, 1 m high under *Populus tremuloides* (1, CELC). **Charlotte Co.**, 10 km NW of New River Beach, 45.2110°N, 66.6170°W, 29.VI-16.VII.2010, R. Webster & C. MacKay, coll. // Old-growth eastern white cedar forest, Lindgren funnel trap (1, AFC). **Kent Co.**, Kouchibouguac National Park, 46.8087°N, 64.9078°W, 12–24.VI.2015, C. Alderson & V. Webster // Poplar/red maple stand, Lindgren funnel trap, 1 m high (1, RWC). **Northumberland Co.**, ca. 2.5 km W of Sevogle, 47.0876°N, 65.8613°W, 26.VI-8.VII.2013, C. Alderson & V. Webster // Old *Pinus banksiana* forest, Lindgren funnel trap (1, RWC). **Queens Co.**, C.F.B. Gagetown, 45.7516°N, 66.1866°W, 2–17.VII.2015, C. Alderson & V. Webster // Old mixed forest with *Quercus rubra*, Lindgren funnel trap 1 m high under trees (1, RWC). **Restigouche Co.**, Dionne Brook P.N.A., 47.9064°N, 68.3441°W, 27.VI-14.VII.2011, M. Roy & V. Webster // Old-growth white spruce & balsam fir forest, Lindgren funnel trap (1, RWC). **York Co.**, 14 km WSW of Tracy S of Rt. 645, 45.6741°N, 66.8661°W, 16–30.VI.2010, R. Webster & C. MacKay, coll. // Old mixed forest with red & white spruce, red & white pine, balsam fir, eastern white cedar, red maple & *Populus* sp., Lindgren funnel trap (1, RWC); same locality data, forest type and trap type but 30.VI-13.VII.2010, R. Webster & K. Burgess (1, RWC).

####### Distribution in Canada and Alaska.

BC, **NB** (Bousquet et al. 2013). All new records of *Cis angustus* Hatch from NB were based on specimens captured in Lindgren funnel traps. This species is widespread (seven localities) in the province but was captured in low numbers at all sites. Adults were captured mostly in conifer-dominated forests and mixed forests. These are the first records of this species from eastern Canada. According to Lawrence (1971), *Cis angustus* is restricted to coniferous forests at higher elevations in the mountains of the Pacific coast of southern BC, south to the southern Sierra Nevada in CA. He mentions further field work might reveal a broader distribution and that this species is part of the Holarctic faunal element. Our records in NB lead to an apparent disjunct distribution of the species in Canada, However, it is likely with additional field work that this species will be found in the intervening areas of northern Canada.

####### Taxonomic notes.

A male was dissected, and its determination as *Cis angustus* was confirmed by John Lawrence. This species was also compared with the closely related Palaearctic species *Cis fagi* Waltl and *Cis fusciclavis* Nyholm, and it was confirmed that *Cis angustus* is distinct from those two species.

###### Cis creberrimus

Taxon classificationAnimaliaColeopteraCiidae

Mellié, 1849

####### Available data.

**New Brunswick, Westmorland Co.**, Moncton, 13.VII.1987, P. Maltais (UMC)

####### Distribution in Canada and Alaska.

ON, QC, NB, NS (Bousquet et al. 2013). *Cis creberrimus* Mellié was first reported from NB by Bousquet et al. (2013) but without supporting data. Chris Majka supplied the data for the specimen, which is in the collection of the University of Moncton. However, the curator of the collection was unable to locate the specimen. The inclusion of this species in the fauna of NB should be considered provisional until the specimen can be located.

####### Taxonomic note.

*Cis creberrimus* is somewhat similar to *Cis angustus* and may be confounded with this species. In view of this, the NB specimen identified as *Cis creberrimus*, could be *Cis angustus*.

###### Cis fuscipes

Taxon classificationAnimaliaColeopteraCiidae

Mellié, 1849 new to New Brunswick

Fig. 5

####### Material examined.

**New Brunswick, Carleton Co.**, Houlton Rd.,16.VI.1978 (no collector given) // on *Populus* sp. 78-2-2548-01 [FIDS #] (10, AFC); Jackson Falls, “Bell Forest”, 46.2210°N, 67.7210°W, 12.VII.2004, K. Bredin, J. Edsall & R. Webster // Mixed forest, on bracket fungi (2, AFC); same locality but 46.2200°N, 67.7231°W, 12–19.VI.2008, R. P. Webster // Rich Appalachian hardwood forest, Lindgren funnel traps (2, AFC); same locality and habitat data but 28.IV-9.V.2009, 14–20.V.2009, 28.V-1.VI.2009, 1–8.VI.2009, R. Webster & M.-A. Giguère // Lindgren funnel traps (7, AFC; 3, CELC); same locality and habitat data but, 8–23.V.2012, 23.V-7.VI.2012, 3–17.VII.2012, 31.VII-14.VIII.2012, 14–29.VIII.2012, C. Alderson & V. Webster // Lindgren funnel traps in canopy of *Acer sacccharum* (3), in canopy of *Fagus grandifolia* (2), in canopy of *Tilia americana* (1) (3, AFC; 3, RWC); Meduxnekeag Valley Nature Preserve, 46.1907°N, 67.6740°W, 23.V-7.VI.2012, C. Alderson & V. Webster // Mixed forest, Lindgren funnel trap in canopy of *Populus tremuloides* (1, RWC). **Charlotte Co.**, 10 km NW of New River Beach, 45.2110°N, 66.6170°W, 31.V-15.VI.2010, R. Webster & C. MacKay // old growth eastern white cedar forest, Lindgren funnel trap (1, AFC). **Gloucester Co.**, Bathurst, Daly Point Nature Preserve, 47.6392°N, 65.6098°W, 13–28.V.2015, 15–25.VI.2015, C. Alderson & V. Webster // Mixed forest, black Lindgren funnel traps 1 m high (2), in canopy (1) (3, AFC). **Kent Co.**, Kouchibouguac National Park, 46.8072°N, 64.9100°W, 12–24.VI.2015, C. Alderson & V. Webster // Jackpine forest, Lindgren funnel trap, 1 m high (1, AFC). **Northumberland Co.**, ca, 2.5 km W of Sevogle, 47.0876°N, 65.8613°W, 11–26.VI.2013, 11–25.VI.2014, C. Alderson & V. Webster // Old *Pinus banksiana* stand, Lindgren funnel traps (1, AFC: 1, RWC); Upper Graham Plains, 47.1001°N, 66.8154°W, 24.VI–9.VII.2014, 9–24.VII.2014, C. Alderson & V. Webster // Old black spruce forest, Lindgren funnel traps (1, AFC; 1, NBM). **Queens Co.**, Scovil, XI.I.1973 (date bolts collected), 8.II.1973 (emergence date), Titus and Newelly // ex. *Ulmus americana* [bolts], 72-2-1696-01C (FIDS #) (28, AFC); Grand Lake Meadows P.N.A., 45.8227°N, 66.1209°W, 19–31.V.2010, R. Webster & C. MacKay // Old silver maple forest with green ash and seasonally flooded marsh, Lindgren funnel traps (2, AFC); same locality data, forest type, and trap but 19.VII-5.VIII.2011, 5–17.VIII.2011, M. Roy & V. Webster (3, CELC; 1, RWC); Jemseg, 45.8412°N, 66.1195°W, 2–14.V.2012, 25.VII-8.VIII.2012, C. Alderson, C. Hughes, & V. Webster // Hardwood woodland near seasonally flooded marsh, Lindgren funnel traps 1 m high under *Quercus macrocarpa* (1, AFC; 1, RWC); Cranberry Lake P.N.A, 46.1125°N, 65.6075°W, 27.V–5.VI.2009, 11–18.VI.2009, 25.VI-1.VII.2009, 15–21.VII.2009, 6–14.VIII.2009, 14–19.VIII.2009, R. Webster & M.-A. Giguère // Mature red oak forest, Lindgren funnel traps (5, AFC; 2, CELC; 2, RWC); C.F.B. Gagetown, 45.7516°N, 66.1866°W, 9–22.V.2013, 22.V–10.VI.2013, C. Alderson & V. Webster // Old mixed forest with *Quercus rubra*, Lindgren funnel traps in canopy of *Quercus rubra* (1, AFC: 1, NBM). **Restigouche Co.**, Dionne Brook P.N.A., 47.9030°N, 68.3503°W, 30.V–15.VI.2011, M. Roy & V. Webster // Old-growth northern hardwood forest, Lindgren funnel traps (2, RWC); Jacquet River Gorge P.N.A., 47.8257°N, 66.0764°W, 15–29.V.2014, 10–25.VI.2014, C. Alderson & V. Webster // Old *Populus balsamifera* stand near river, Lindgren funnel traps in canopy of *Populus balsamifera* (1, AFC, 1, NBM); ca. 3 km SE of Simpsons Field, 47.5277°N, 66.5142°W, 14–28.V.2015, 28.V-16.VI.2015, C. Alderson & V. Webster // Old cedar & spruce forest with *Populus balsamifera* & *Populus tremuloides*, Lindgren funnel traps (3, AFC). **Sunbury Co.**, Acadia Research Forest, 45.9866°N, 66.3441°W, 25.V-2.VI.2009, 2–9.VI.2009, 9–16.VI.2009, 4–11.VIII.2009, R. Webster & M.-A. Giguère // Red spruce forest with red maple & balsam fir, Lindgren funnel traps (3, AFC; 1, CELC; 1, RWC); Gilbert Island, 45.8770°N, 66.2954°W, 18–28.V.2012, 25.VII-8.VIII.2012, 8–21.VIII.2012, 13–23.V.2013, C. Alderson, C. Hughes, & V. Webster // Hardwood forest, Lindgren funnel traps in canopy of *Juglans cinerea* (1), in canopy of *Populus tremuloides* (1), 1 m high under *Juglans cinerea* (3) (2, AFC; 1, CELC; 1, NBM; 1, RWC); same locality data, forest type but 21.VIII-7.IX.2012, C. Hughes & K. Van Rooyen // Lindgren funnel trap 1 m high under *Juglans cinerea* (1, RWC). **York Co.**, 15 km W of Tracy off Rt. 645, 45.6848°N, 66.8821°W, 8–15.VI.2009, 15–21.VI.2009, 20–29.VII.2009, R. Webster & M.-A. Giguère // Old red pine forest, Lindgren funnel traps (2, AFC; 3, RWC); same locality and habitat data but 10–26.V.2010, 26.V-4.VI.2010, R. Webster & C. MacKay // Old red pine forest, Lindgren funnel traps (3, AFC; 1, RWC); Fredericton, Odell Park, 45.9539°N, 66.6666°W, 2–15.V.2013, 27.V-10.VI.2013, C. Alderson & V. Webster // Hardwood stand, Lindgren funnel traps in canopy (1, AFC; 1, NBM); Douglas, Currie Mountain, 45.9832°N, 66.7564°W, 3–15.V.2013, 27.V-10.VI.2013, C. Alderson & V. Webster // Old *Pinus strobus* stand, Lindgren funnel trap in canopy of *Pinus strobus* (1), 1 m high under *Pinus strobus* (1) (2, AFC); Douglas, Currie Mountain, 45.9844°N, 66.7592°W, 3–15.V.2013, C. Alderson & V. Webster // Mixed forest with *Quercus rubra*, Lindgren funnel trap in canopy of *Quercus rubra* (1), 1 m high under *Quercus rubra* (1) (1, AFC, 1, NBM); Eel River P.N.A., 45.8966°N, 67.6345°W, 21.V–2.VI.2014, 2–20.VI.2014, 20.VI–2.VII.2014, 28.VII–12.VIII.2014, C. Alderson & V. Webster // Old-growth eastern white cedar swamp/fen, Lindgren funnel traps (2, AFC; 3, NBM); Keswick Ridge, 45.9962°N, 66.8781°W, 22.V-4.VI.2014, 18–30.VII.2014, 30.VII-13.VIII.2014, C. Alderson & V. Webster // Mixed forest, Lindgren funnel traps in canopy (3, AFC).

####### Distribution in Canada and Alaska.

NT, BC, AB, SK, MB, ON, QC, **NB**, NS, NF (Bousquet et al. 2013). Nearly all of the new records of *Cis fucipes* Mellié in NB were based on specimens captured in Lindgren funnel traps. This species is widespread (25 sites) and the most common ciid in the province, being captured in traps at nearly all sites and forest types where Lindgren traps were used.

###### Cis horridulus

Taxon classificationAnimaliaColeopteraCiidae

Casey, 1898 new to New Brunswick

Fig. 6

####### Material examined.

**New Brunswick, Carleton Co.**, Jackson Falls, “Bell Forest”, 46.2200°N, 67.7231°W, 1–8.VI.2009, R. Webster & M.-A. Giguère // Rich Appalachian hardwood forest with some conifers, Lindgren funnel trap (1, RWC). **Gloucester Co.**, Bathurst, Daly Point Nature Preserve, 47.6392°N, 65.6098°W, 15–25.VI.2015, C. Alderson & V. Webster // Mixed forest, green Lindgren funnel trap 1 m high under trees (1, AFC). **Kent Co.**, Kouchibouguac National Park, 46.8087°N, 64.9078°W, 27.V-12.VI.2015, C. Alderson & V. Webster // Poplar/red maple stand, Lindgren funnel trap, 1 m high (1, AFC); same locality but 46.8072°N, 64.9100°W, 27.V–12.VI.2015, C. Alderson & V. Webster // Jackpine forest, Lindgren funnel trap, 1 m high (1, AFC). **Northumberland Co.**, ca. 2.5 km W of Sevogle, 47.0876°N, 65.8613°W, 11–26.VI.2013, 27.V–11.VI.2014, C. Alderson & V. Webster // Old *Pinus banksiana* stand, Lindgren funnel traps (1, AFC, 2, RWC); Upper Graham Plains, 47.1001°N, 66.8154°W, 10–24.VI.2014, 24.VI–9.VII.2014, C. Alderson & V. Webster // Old black spruce forest, Lindgren funnel traps (1, AFC; 1, NBM). **Queens Co.**, Cranberry Lake P.N.A., 46.1125°N, 65.6075°W, 21–27.V.2009, R.P. Webster & M.-A. Giguère, coll. // Old red oak forest, Lindgren funnel trap (1, AFC); C.F.B. Gagetown, 45.7516°N, 66.1866°W, 22.V-4.VI.2013, C. Alderson & V. Webster // Old mixed forest with *Quercus rubra*, Lindgren funnel traps in canopy of *Quercus rubra* (3, RWC). **York Co.**, 15 km W of Tracy off Rt. 645, 45.6848°N, 66.8821°W, 1–8.VI.2009, R.P. Webster & M.-A. Giguère // Old red pine forest, Lindgren funnel trap (1, RWC); same locality, forest type, and trap type but 10–26.V.2010, 18.V–2.VII.2010, R. Webster & C. MacKay (1, AFC; 1, CELC; 1, RWC); 14 km WSW of Tracy S of Rt. 645, 45.6741°N, 66.8661°W, 10–26.V.2010, 26.V–2.VI.2010, R. Webster & C. MacKay, coll. // Old mixed forest with red & white spruce, red & white pine, balsam fir, eastern white cedar, red maple & *Populus* sp., Lindgren funnel traps (1, AFC; 1, CELC; 4, RWC).

####### Distribution in Canada and Alaska.

NT, BC, MB, ON, QC, **NB**, NS (Bousquet et al. 2013). All new records of *Cis horridulus* Casey from NB were provided by specimens captured in Lindgren funnel traps. This species is widespread (10 localities) in the province. Adults were captured in hardwood, mixed, and conifer forests.

####### Taxonomic notes.

A male was dissected, and its genitalia compared with those of the closely related Palaearctic species *Cis punctulatus* Gyllenhal and *Cis tomentosus* Mellié, and it was confirmed that *Cis horridulus* is not conspecific with these two species.

###### Cis levettei

Taxon classificationAnimaliaColeopteraCiidae

(Casey, 1898)

Fig. 7

####### Material examined.

**New Brunswick, Albert Co.**, Fundy N.P., Point Wolfe R. Trail, 25.VII.1968, E.E. Lindquist, Ex: bracket fungi (99, CNC); Caledonia Gorge P.N.A., 45.8380°N, 64.8484°W, 3.VII.2011, R.P. Webster // near Turtle Creek, Old-growth sugar maple & yellow birch forest, under bark of sugar maple log (1, RWC); same data as previous but R. Webster & A. Fairweather // in *Polyporus varius* on side of log (1, NBM). **Carleton Co.**, Jackson Falls, “Bell Forest”, 46.2200°N, 67.7231°W, 8–16.VI.2009, 16–21.VI.2009, R. Webster & M.-A. Giguère // Rich Appalachian hardwood forest with some conifers, Lindgren funnel trap (1, AFC; 1, RWC); same locality data, forest type, and collectors but 7.VII.2009 // in polypore fungi on log (1, RWC); Meduxnekeag Valley Nature Preserve, 46.1896°N, 67.6700°W, 25.VI.2007, R.P. Webster // Hardwood forest, in polypore fungi (2, CELC; 3, RWC). **Kent Co.**, Kouchibouguac, N.P., 7.VII.1977, J.R. Vockeroth, Code-5584T (5, CNC). **Northumberland Co.**, ca. 2.5 km W of Sevogle, 47.0876°N, 65.8613°W, 28.V–11.VI.2013, 11–26.VI.2013, C. Alderson & V. Webster // Old *Pinus banksiana* stand, Lindgren funnel traps (1, AFC; 1, RWC); Upper Graham Plains, 47.1001°N, 66.8154°W, 24.VI–9.VII.2014, C. Alderson & V. Webster // Old black spruce forest, Lindgren funnel trap (1, AFC). **Restigouche Co.**, Dionne Brook P.N.A., 47.9030°N, 68.3503°W, 27.VI-14.VII.2011, M. Roy & V. Webster // Old-growth northern hardwood forest, Lindgren funnel trap (1, RWC); same locality data and forest type but 30.V–15.VI.2011 (1, CELC), and 27.VI.2011, R.P. Webster, J. Sweeney, & M. Turgeon // in old polypore fungi on rotten log (1, RWC); ca. 3 km SE of Simpsons Field, 47.5277°N, 66.5142°W, 28.V-15.VI.2015, C. Alderson & V. Webster // Old cedar & spruce forest with *Populus balsamifera* & *Populus tremuloides*, Lindgren funnel traps (2, AFC). **Sunbury Co.**, Acadia Research Forest, 46.0188°N, 66.3765°W, 17.VIII.2007, R.P. Webster // Road 16 Control, Mature red spruce & red maple forest, inside *Piptoporus betulinus* (birch polypore) (1, AFC; 1, CELC; 3, RWC); Grand Lake Meadows P.N.A., off Coy Rd., 45.9838°N, 66.1925°W, 15.VI.2013, Amanda Bremner // On *Fomes fomertarius* (1, NBM). **York Co.**, 14 km WSW of Tracy S of Rt. 645, 45.6741°N, 66.8661°W, 2–16.VI.2010, R. Webster & C. MacKay, coll. // Old mixed forest with red & white spruce, red & white pine, balsam fir, eastern white cedar, red maple & *Populus* sp., Lindgren funnel trap (1, AFC); 15 km W of Tracy off Rt. 645, 45.6848°N, 66.8821°W, 10–26.V.2010, R. Webster & C. MacKay, coll. // Old red pine forest, Lindgren funnel trap (1, AFC).

####### Distribution in Canada and Alaska.

BC, AB, SK, MB, ON, QC, NB, NS, PE, NF (Bousquet et al. 2013). Most records of *Cis levettei* (Casey) in NB were from specimens captured in Lindgren funnel traps. This species is widespread (13 localities) and fairly common in the province, occurring in hardwood, mixed, and conifer forests. Specimens with habitat data were found in *Piptoporus betulinus*, *Fomes fomertarius* (L.) Fr., *Polyporus varius* (Pers.) Fr., and bracket fungi. This species was first reported from NB by McNamara (1991) but without supporting data (many specimens in CNC that are reported here).

####### Taxonomic notes.

A male was dissected, and its genitalia compared with those of the closely related Palaearctic species *Cis castaneus* (Herbst), *Cis glabratus* Mellié, *Cis hanseni* Strand, *Cis jacquemartii* Mellié, and *Cis lineatocribatus* Mellié, and it was confirmed that *Cis levettei* is not conspecific to any of these.

###### Cis striatulus

Taxon classificationAnimaliaColeopteraCiidae

Mellié, 1849* new to New Brunswick

Fig. 8

Cis flavipes Lucas, 1847: 470 (not Cis flavipes Motschulsky, 1845); Abeille de Perrin 1874: 33 (syn.)
Cis fraterna Casey, 1898: 80, **new synonym**; Lawrence 1971: 475 (as syn. of Cis striolatus Casey)
Cis fraternus ; Abdullah 1973: 214 (mandatory change to agree in gender)
Cis macilentus Casey, 1898: 80, **new synonym**; Lawrence 1971: 475 (as syn. of Cis striolatus Casey)
Cis macilentus ; Abdullah 1973: 218 (mandatory change to agree in gender)
Cis peyronis Abeille de Perrin, 1874: 65; Abeille de Perrin 1876: 311 (syn.)
Cis striolata Casey, 1898: 79, **new synonym**
Cis striolatus ; Lawrence 1971: 475, Abdullah 1973: 226 (mandatory change to agree in gender)

####### Material examined.

**New Brunswick, Carleton Co.**, Meduxnekeag Valley Nature Preserve, 46.1907°N, 67.6740°W, 7–21.VI.2012, C. Alderson & V. Webster. // Old mixed forest, Lindgren funnel trap in canopy of *Populus tremuloides* (1, AFC). **Charlotte Co.**, 10 Km NW of New River Beach, 45.2110°N, 66.6170°W, 17–31.V.2010, R. Webster & C. MacKay (1, CELC), **Gloucester Co.**, Bathurst, Daly Point Nature Preserve, 47.6392°N, 65.6098°W, 28.V-15.VI.2015, 25.VI-9.VII.2015, C. Alderson & V. Webster // Mixed forest, green Lindgren funnel traps in canopy (5) black Lindgren funnel traps in canopy (2) (7, AFC). **Kent Co.**, Kouchibouguac National Park, 46.8072°N, 64.9100°W, 24.VI-7.VII.2015, C. Alderson & V. Webster // Jackpine forest, Lindgren funnel trap, 1 m high (1, AFC). **Northumberland Co.**, ca. 1.5 km NW of Sevogle, 47.0939°N, 65.8387°W, 11–26.VI.2013, 26.VI-8.VII.2013, C. Alderson & V. Webster // *Populus tremuloides* stand with a few conifers, Lindgren funnel traps in canopy of *Populus tremuloides* (1, AFC; 1 NBM; 1, RWC); ca. 2.5 km NW of Sevogle, 47.0879°N, 65.8585°W, 10–25.VI.2014, C. Alderson & V. Webster // Old *Pinus banksiana* forest, Lindgren funnel trap (1, RWC); Upper Graham Plains, 47.1001°N, 66.8154°W, 28.V-10.VI.2014, C. Alderson & V. Webster // Old black spruce forest, Lindgren funnel traps (1, AFC; 1, RWC). **Queens Co.**, Cranberry Lake P.N.A., 46.1125°N, 65.6075°W, 21–27.V.2009, R. Webster & M.-A. Giguère // Red oak forest, Lindgren funnel trap (1, RWC); same locality data and forest type but 22–29.VI.2011, 4–18.VIII.2011, M. Roy & V. Webster // Lindgren funnel traps (2, RWC); C.F.B. Gagetown, 45.7516°N, 66.1866°W, 17.VI-3.VII.2013, C. Alderson & V. Webster // Old mixed forest with *Quercus rubra*, Lindgren funnel traps in canopy of *Quercus rubra* (1, AFC: 1, NBM). **Restigouche Co.**, Jacquet River Gorge P.N.A., 47.8257°N, 66.0764°W, 10–25.VI.2014, C. Alderson & V. Webster // Old *Populus balsamifera* stand near river, Lindgren funnel trap in canopy of *Populus balsamifera* (1, NBM); same locality data and forest type, but 9–22.VII.2014 (1, CELC). **Sunbury Co.**, Acadia Research Forest, 45.9866°N, 66.3441°W, 16–24.VI.2009, 8–13.VII.2009, R. Webster & M.-A. Giguère // Red spruce forest with red maple & balsam fir, Lindgren funnel traps (3, RWC); Gilbert Island, 45.8770°N, 66.2954°W, 28.V-12.VI.2012, 12–29.VI.2012, 29.VI-11.VII.2012, 25.VII-8.VIII.2012, 8–21.VIII.2012, 20.VI-5.VII.2013, 5–17.VII.2013, C. Alderson, C. Hughes, & V. Webster // Hardwood forest, Lindgren funnel traps in canopy of *Populus tremuloides* (7), in canopy of *Tilia americana* (1) (4, AFC; 1, CELC; 2, NBM; 1, RWC). **York Co.**, 16 km W of Tracy off Rt. 645, 45.6855°N, 66.8847°W, 18.V–2.VI.2010, 16–30.VI.2010, R. Webster & C. MacKay // Old red pine forest, Lindgren funnel traps (1, AFC; 1, RWC); 15 km W of Tracy off Rt. 645, 45.6848°N, 66.8821°W, 19–25.V.2009, R. Webster & M.-A. Giguère, coll. // Old red pine forest, Lindgren funnel trap (1, AFC); Keswick Ridge, 45.9962°N, 66.8781°W, 4–19.VI.2014, C. Alderson & V. Webster // Mixed forest, Lindgren funnel trap in canopy (1, AFC); Eel River P.N.A., 45.8966°N, 67.6345°W, 21.V–2.VI.2014, 2–20.VI.2014, 20.VI–2.VII.2014, C. Alderson & V. Webster // Old-growth eastern white cedar swamp/fen, Lindgren funnel traps (2, AFC; 2, NBM).

####### Distribution in Canada and Alaska.

NT, BC, AB, MB, ON, QC, **NB**, NS (Bousquet et al. 2013). All new records of *Cis striatulus* Mellié from NB were based on specimens captured in Lindgren funnel traps. This species is widespread (16 localities) and fairly common in the province, occurring in hardwood, mixed, and conifer forests.

####### Taxonomic notes.

A drawing of the male tegmen of the North American *Cis striolatus* Casey was provided by Lawrence (1971), who also commented that the species would be a synonym of *Cis striatulus*, the latter occurring in Central Europe, the Caucasus, and northern Africa. The type locality of *Cis striolatus* is Salida (Colorado). Although no specimen collected near the type locality was examined, the identification of the species is confident, and sufficient data on its morphological limits were provided by Lawrence (1971).The type locality of *Cis striatulus* is southern France, and there were specimens from localities in France, Germany, and northern Iran (the Caucasus) available for comparison. A few males were dissected, confirming that tegmen shape is the same as shown in Lawrence (1971). External morphological features and known intraspecific variation matched between Nearctic and Palaeartic populations of *Cis striolatus* and *Cis striatulus*, respectively, so we propose their synomymization here. *Cis striolatus* had two junior synonyms previously proposed by Lawrence (1971): *Cis fraternus* Casey and *Cis macilentus* Casey. These names refer to variations of *Cis striolatus*, and thus, we also propose them as new synonymies of *Cis striatulus*. As *Cis flavipes* Lucas, 1847 is a junior homonym of *Cis flavipes* Motschulsky, 1845, the oldest available name becomes *Cis striatulus* Mellié, 1849.

###### Cis submicans

Taxon classificationAnimaliaColeopteraCiidae

Abeille de Perrin, 1874*

Fig. 9

Cis submicans Abeille de Perrin, 1874: 28 (as a variety of Cis setiger Mellié, 1849)
Cis micans : auctt. (non Fabricius, 1792; see Jelínek 2007)
Cis pistoria Casey, 1898: 79, **new synonym**
Cis pistorius ; Abdullah 1973: 221 (unjustified emendation)

####### Material examined.

**New Brunswick, Carleton Co.**, Meduxnekeag Valley Nature Preserve, 46.1907°N, 67.6740°W, 7.IX.2004, R.P. Webster (1, RWC); Jackson Falls, “Bell Forest”, 46.2200°N, 67.7231°W, 9.X.2006, R.P. Webster // Rich Appalachian hardwood forest with some conifers, under bark of fallen beech log covered with polypore fungi (2, CELC; 4, RWC); same locality data and forest type but 23–28.IV.2009, R. Webster & M.-A. Giguère // Lindgren funnel trap (1, AFC). **Gloucester Co.**, Bathurst, Daly Point Nature Preserve, 47.6392°N, 65.6098°W, 13–28.V.2015, C. Alderson & V. Webster // Mixed forest, Lindgren funnel trap 1 m high under trees (1, RWC). **Kent Co.**, Kouchibouguac National Park, 46.8087°N, 64.9078°W, 21–27.V.2015, C. Alderson & V. Webster // Poplar/red maple stand, Lindgren funnel trap, 1 m high (1, AFC). **Queens Co.**, Grand Lake near Scotchtown, 45.8762°N, 66.1816°W, 19.IX.2006, R.P. Webster // Oak & maple forest, under bark of oak (1, CELC; 1, RWC); Cranberry Lake P.N.A., 46.1125°N, 65.6075°W, 24.IV–5.V.2009, R.P. Webster & M.-A. Giguère, coll. // Old red oak forest, Lindgren funnel traps (1, CELC; 2, AFC). **Restigouche Co.**, Jacquet River Gorge P.N.A., 47.8257°N, 66.0764°W, 15–29.V.2014, C. Alderson & V. Webster // Old *Populus balsamifera* stand near river, Lindgren funnel traps 1 m high under trees (1, NBM, 3, RWC); ca. 3 km SE of Simpsons Field, 47.5277°N, 66.5142°W, 14–28.V.2015, C. Alderson & V. Webster // Old cedar & spruce forest with *Populus balsamifera* & *Populus tremuloides*, Lindgren funnel trap (1, AFC). **York Co.**, Charters Settlement, 45.8395°N, 66.7391°W, 6.V.2008, R.P. Webster // Mixed forest, in flight, collected with net between 15:00 & 17:00 h (1, RWC).

####### Distribution in Canada and Alaska.

NT, AB, SK, MB, ON, QC, NB, NS (Bousquet et al. 2013). Most records of *Cis submicans* from NB were based on specimens captured in Lindgren funnel traps. This species is widespread (nine localities) and fairly common in the province, with most records from hardwood-dominated and mixed forests. Some specimens were found under bark of a log covered with polypore fungi. *Cis submicans* (as *Cis pistoria* Casey) was first reported from NB by Majka (2007) on the basis of a specimen from the Meduxnekeag Valley Nature Preserve (record is reported here).

####### Taxonomic notes.

*Cis pistoria* is the only New World member of the *Cis boleti* species group, which also includes the Palaearctic *Cis boleti* (Scopoli), *Cis micans* (Fabricius), *Cis polypori* Chûjô (also treated as a subspecies of *Cis boleti*), *Cis rugulosus* Mellié and *Cis submicans* Abeille de Perrin (Chûjô 1939, Lawrence 1971). Specimens of all these species and subspecies were available for comparison and, among them, aedeagi of *Cis pistoria* from North America and those of *Cis submicans* were indiscernible. *Cis pistoria* was described by Casey (1898) based on specimens from “Rhode Island (Boston Neck)” in USA, a locality on the northeastern coast and about 500 linear km south of NB. Most reports of *Cis pistoria* to date were in northeastern USA and in Canada (Lawrence 1971, Bousquet et al. 2013). *Cis submicans* was described by Abeille de Perrin (1874) as a variety of *Cis setiger* Mellié (currently *Cis villosulus*) and based on specimens from the Caucasus and Poland. It is important to note that most *Cis submicans* available for comparison were also from northern Iran (in the Caucasus) and Poland. As specimens currently recognized as *Cis pistoria* fits well in the morphological limits of the Palaearctic *Cis submicans*, we propose their synonymization here. *Cis submicans* has a Holarctic distribution, occurring in Europe and the Caucasus, and in northeastern North America.

###### Dolichocis laricinus

Taxon classificationAnimaliaColeopteraCiidae

(Mellié, 1849)* new to New Brunswick

Fig. 10

Ennearthron laricinum Mellié, 1849: 355, pl. 12, fig. 3
Dolichocis indistinctus Hatch, 1962: 234, **new synonym**

####### Material examined.

**New Brunswick, Kent Co.**, Kouchibouguac National Park, 46.8072°N, 64.9100°W, 12–14.VI.2015, 7–22.VII.2015, C. Alderson & V. Webster // Jackpine forest, Lindgren funnel traps, 1 m high (1, AFC; 1, RWC). **Northumberland Co.**, ca, 2.5 km W of Sevogle, 47.0876°N, 65.8613°W, 28.V–11.VI.2013, 11–26.VI.2013, 10–24.VI.2014, 24.VI–9.VII.2014, C. Alderson & V. Webster // Old *Pinus banksiana* stand, Lindgren funnel traps (1, CELC; 4, RWC). **York Co.**, Keswick Ridge, 45.9962°N, 66.8781°W, 19.V–3.VI.2015, C. Alderson & V. Webster // Mixed forest, Lindgren funnel trap 1 m high under trees (1, RWC).

####### Distribution in Canada and Alaska.

BC, QC, **NB** (Bousquet et al. 2013). All new records of *Dolichocis laricinus* (Mellié) from NB were based on specimens captured in Lindgren funnel traps. This species is currently known from three localities in NB. Specimens were captured in jack pine forests (*Pinus banksiana* Lamb.) at two localities and a mixed forest. These are the first records of this species from the Maritime Provinces.

####### Taxonomic notes.

The genus *Dolichocis* Dury has only four species: *Dolichocis indistinctus* Hatch and *Dolichocis manitoba* Dury from North America, the Eurasian *Dolichocis laricinus* (Mellié), and *Dolichocis yuasai* (Chûjô) from Japan. The possible synonomy of *Dolichocis indistinctus* Hatch and *Dolichocis laricinus* was first proposed by Lawrence (1971). The type locality of *Dolichocis indistinctus* (Stanley, BC) is close to the northwestern coast, but the species has a broad distribution in North America, and there is currently no doubt about its identification. The type locality of *Dolichocis laricinus* is Paris (France), and specimens from Poland and France were available for comparison. A male *Dolichocis indistinctus* from NB was dissected, and its aedeagus is exactly the same as in European specimens of *Dolichocis laricinus*. Specimens of *Dolichocis manitoba* were also examined and dissected, confirming that its male genitalia are quite distinct from those of *Dolichocis indistinctus* and *Dolichocis laricinus*. Unfortunately, no *Dolichocis yuasai* were available, and no opinion can be given on this species. As *Dolichocis indistinctus* is well within the morphological limits of *Dolichocis laricinus*, we propose their synonymy. The species has a Holarctic distribution. In North America, it occurs on the same host fungi of *Dolichocis manitoba* Dury, but *Dolichocis laricinus* appears to be much rarer (Lawrence 1971).

###### Dolichocis manitoba

Taxon classificationAnimaliaColeopteraCiidae

Dury, 1919

Fig. 11

####### Material examined.

**New Brunswick, Albert Co.**, Fundy N.P., Point Wolfe R. Trail, 25.VII.1968, E.E. Lindquist, Ex: bracket fungi (8, CNC). **Northumberland Co.**, Ludlow, 18.VII.1967, D. P. Pielou, Ex: *Polyporus betulinus*, H-21 (91, CNC); Taxis [River], 26.V.1967, D.P. Pielou, Ex: *Polyporus betulinus*, G-364 (750, CNC); ca. 2.5 km W of Sevogle, 47.0876°N, 65.8613°W, 9–23.VII.2014, C. Alderson & V. Webster // Old *Pinus banksiana* stand, Lindgren funnel trap (1, RWC). **Queens Co.**, Jemseg, 17.V.1967 (H245), D.P. Pielou, Ex: *Polyporus betulinus* (1, CNC); Grand Lake Meadows P.N.A., 45.8227°N, 66.1209°W, 21.VI–5.VII.2011, M. Roy & V. Webster // Old silver maple forest with green ash and seasonally flooded marsh, Lindgren funnel trap (1, RWC). **Restigouche Co.**, Matapedia, D.P. Pielou (64, CNC); ca. 3 km SE of Simpsons Field, 47.5277°N, 66.5142°W, 16–25.VI.2015, C. Alderson & V. Webster // Old cedar & spruce forest with *Populus balsamifera* & *Populus tremuloides*, Lindgren funnel trap (1, AFC). **Sunbury Co.**, Wirral, 4.VII.1967 (H414), D.P. Pielou, Ex: *Polyporus betulinus* (1, CNC); Acadia Research Forest, 45.9866°N, 66.3441°W, 24–30.VI.2009, R. Webster & M.-A. Giguère // Red spruce forest with red maple & balsam fir, Lindgren funnel trap (1, CELC; 1, RWC). **York Co.**, 15 km W of Tracy off Rt. 645, 45.6848°N, 66.8821°W, 21–28.VI.2009, R.P. Webster & M.-A. Giguère // Old red pine forest, Lindgren funnel trap (1, RWC); same locality, forest type, and trap type but 14–20.VII.2009 (1, CELC), and 16–30.VI.2010, R. Webster & C. MacKay (1, CELC; 2, RWC); 14 km WSW of Tracy S of Rt. 645, 45.6741°N, 66.8661°W, 10–26.V.2010, 16–30.VI.2010, 30.VI–13.VII.2010, R. Webster & C. MacKay, coll. // Old mixed forest with red & white spruce, red & white pine, balsam fir, eastern white cedar, red maple & *Populus* sp., Lindgren funnel traps (1, AFC; 3, RWC); Fredericton, Odell Park, 45.9484°N, 66.6802°W, 17.VI–3.VII.2014, 17.VII–1.VIII.2014, C. Alderson & V. Webster // Old mixed forest, Lindgren funnel trap in front of tree hole (2, RWC).

####### Distribution in Canada and Alaska.

NT, BC, AB, MB, ON, QC, NB (Bousquet et al. 2013). Most records of *Dolichocis manitoba* from NB were based on specimens captured in Lindgren funnel traps. This species is widespread (12 localities) and fairly common in the province. This species was first reported from NB by Pielou and Verna (1968) (records included above). *Dolichocis manitoba* was captured in hardwood, mixed, and conifer forests. Specimens were reared from *Piptoporus betulinus* (=*Polyporus betulinus*) from four sites by D.P. Pielou.

###### Hadreule elongatula

Taxon classificationAnimaliaColeopteraCiidae

(Gyllenhal, 1827)†

Fig. 12

####### Material examined.

**New Brunswick, Northumberland Co.**, Ludlow, 6.VI-31.VII.1967, 21.VI.1968, 2.VII.1968, D. P. Pielou, Ex: *Polyporus betulinus* (24, CNC); ca. 2.5 km W of Sevogle, 47.0876°N, 65.8613°W, 28.V–11.VI.2013, 11–26.VI.2013, C. Alderson & V. Webster // Old *Pinus banksiana* stand, Lindgren funnel traps (2, AFC; 1, RWC); Upper Graham Plains, 47.1001°N, 66.8154°W, 24.VI–9.VII.2014, 9–24.VII.2014, C. Alderson & V. Webster // Old black spruce forest, Lindgren funnel traps (3, AFC; 1, NBM). **Queens Co.**, Castaway Brook, 5.VII.1968, D.P. Pielou, Ex: *Polyporus betulinus*, H-129 (1, CNC). **Restigouche Co.**, Jacquet River Gorge P.N.A., 47.8257°N, 66.0764°W, 25.VI–9.VII.2014, C. Alderson & V. Webster // Old *Populus balsamifera* stand near river, Lindgren funnel trap in canopy of *Populus balsamifera* (1, NBM). **Sunbury Co.**, Acadia Research Forest, 45.9866°N, 66.3441°W, 19–25.V.2009, 2–9.VI.2009, 9–16.VI.2009, 16–24.VI.2009, 24–30.VI.2009, R.P. Webster & M.-A. Giguère // Red spruce forest with red maple & balsam fir, Lindgren funnel traps (3, CELC; 2, AFC; 7, RWC). **York Co.**, 15 km W of Tracy off Rt. 645, 45.6848°N, 66.8821°W, 15–21.VI.2009, R.P. Webster & M.-A. Giguère // Old red pine forest, Lindgren funnel traps (2, RWC); same locality data, forest type, and trap type but 16–30.VI.2010, R. Webster & C. MacKay (1, AFC).

####### Distribution in Canada and Alaska.

NB (Bousquet et al. 2013). *Hadreule elongatula* (Gyllenhal) is widespread throughout Europe, Siberia, and North Africa (Lawrence 1971). Lawrence (1971) hypothesized that the species would have a broader distribution in North America, but in the subsequent decades, it has been found only in NB, where it was first reported by Pielou and Verna (1968) from specimens reared from *Piptoporus betulinus* (=*Polyporus betulinus*) at two localities (reported above). It was probably introduced from Europe (Lawrence 1971). All recent records of this species from NB were based on specimens captured in Lindgren funnel traps. This species is widespread (seven localities) and fairly common in the province. This adventive species was captured mostly in conifer forests in NB.

####### Taxonomic notes.

It is worth mentioning that the correct spelling of the genus name is *Hadreule*, not *Hadraule* (see Orledge and Booth 2006). In several works, the publication date of this genus, described by Thomson, was cited as being 1863. In Thomson (1863) the spelling is “*Hadraule*”. However, the name was indeed proposed four years before by the same author, but spelled as “*Hadreule*” (Thomson 1859: 91). Therefore, “*Hadraule*” is an incorrect subsequent spelling.

###### Malacocis brevicollis

Taxon classificationAnimaliaColeopteraCiidae

(Casey, 1898) new to New Brunswick

Fig. 13

####### Material examined.

**New Brunswick, Carleton Co.**, Jackson Falls, “Bell Forest”, 46.2200°N, 67.7231°W, 12–19.VI.2008, 19–27.VI.2008, R. Webster // Rich Appalachian hardwood forest with some conifers, Lindgren funnel traps (1, AFC; 1, NBM); same locality and habitat data but 21–28.VI.2009, R. Webster & M.-A. Giguère // Lindgren funnel traps (1, AFC; 1, RWC). **Gloucester Co.**, Bathurst, Daly Point Nature Preserve, 47.6392°N, 65.6098°W, 25.VI-9.VII.2015, C. Alderson & V. Webster // Mixed forest, green Lindgren funnel trap 1 m high (1), black Lindgren funnel trap 1 m high (1) (2, AFC). **Kent Co.**, Kouchibouguac, N.P., 2.VII.1977, J.R. Vockeroth, Code-5466-F (1, CNC); Kouchibouguac National Park, 46.8087°N, 64.9078°W, 24.VI-7.VII.2015, C. Alderson & V. Webster // Poplar/red maple stand, Lindgren funnel trap, 5 m high (1, AFC). **Northumberland Co.**, ca. 1.5 km NW of Sevogle, 47.0939°N, 65.8387°W, 11–26.VI.2013, C. Alderson & V. Webster // *Populus tremuloides* stand with a few conifers, Lindgren funnel trap 1 m high under *Populus tremuloides* (1, AFC). **Queens Co.**, Cranberry Lake P.N.A., 46.1125°N, 65.6075°W, 15–21.VII.2009, R.P. Webster & M.-A. Giguère, coll. // Old red oak forest, Lindgren funnel trap (1, RWC); same locality, forest and trap type but 13–20.VII.2011, M. Roy & V. Webster (1, CELC); Grand Lake Meadows P.N.A., 45.8227°N, 66.1209°W, 29.VI–12.VII.2010, R. Webster, C. MacKay, M. Laity & R. Johns // Old silver maple forest with green ash and seasonally flooded marsh, Lindgren funnel trap (1, RWC); C.F.B. Gagetown, 45.7516°N, 66.1866°W, 17.VI-3.VII.2013, C. Alderson & V. Webster // Old mixed forest with *Quercus rubra*, Lindgren funnel trap in canopy of *Quercus rubra* (1, AFC). **Restigouche Co.**, Dionne Brook P.N.A., 47.9064°N, 68.3441°W, 27.VI–14.VII.2011, M. Roy & V. Webster // Old-growth white spruce & balsam fir forest, Lindgren funnel traps (3, RWC); ca. 3 km SE of Simpsons Field, 47.5277°N, 66.5142°W, 25.VI–10.VII.2015, C. Alderson & V. Webster // Old cedar & spruce forest with *Populus balsamifera* & *Populus tremuloides*, Lindgren funnel trap (1, AFC). **Sunbury Co.**, Acadia Research Forest, 45.9866°N, 66.3441°W, 16–24.VI.2009, 24–30.VI.2009, R.P. Webster & M.-A. Giguère // Red spruce forest with red maple & balsam fir, Lindgren funnel traps (2, RWC). **York Co.**, 15 km W of Tracy off Rt. 645, 45.6848°N, 66.8821°W, 21–28.VI.2009, R.P. Webster & M.-A. Giguère // Old red pine forest, Lindgren funnel trap (1, RWC); 14 km WSW of Tracy S of Rt. 645, 45.6741°N, 66.8661°W, 16–30.VI.2010, R. Webster & C. MacKay, coll. // Old mixed forest with red & white spruce, red & white pine, balsam fir, eastern white cedar, red maple & *Populus* sp., Lindgren funnel trap (1, RWC); same locality data, forest type, and trap type but 30.VI-13.VII.2010, R. Webster & K. Burgess (1, RWC); Fredericton, Odell Park, 45.9539°N, 66.6666°W, 24.VI–9.VII.2013, C. Alderson & V. Webster // Hardwood stand, Lindgren funnel trap in canopy (1, AFC); Fredericton, U.N.B. Woodlot, 45.9206°N, 66.6520°W, 14–27.VI.2013, C. Alderson & V. Webster // Mature mixed forest, Lindgren funnel trap 2 m high (1, AFC); Eel River P.N.A., 45.8966°N, 67.6345°W, 2–15.VII.2014, C. Alderson & V. Webster // Old-growth eastern white cedar swamp/fen, Lindgren funnel trap (1, NBM).

####### Distribution in Canada and Alaska.

MB, ON, QC, **NB**, NS, NF (Bousquet et al. 2013). All but one of the new records of *Malacocis brevicollis* (Casey) from NB were based on specimens captured in Lindgren funnel traps. This species is widespread (15 localities) in NB but was captured in low numbers at each site. Specimens were captured in hardwood, mixed, and conifer forests.

###### Orthocis punctatus

Taxon classificationAnimaliaColeopteraCiidae

(Mellié, 1849) new to New Brunswick

Fig. 14

####### Material examined.

**New Brunswick, Carleton Co.**, Jackson Falls, “Bell Forest”, 46.2200°N, 67.7231°W, 9–14.V.2009, R. Webster & M.-A. Giguère // Rich Appalachian hardwood forest with some conifers, Lindgren funnel trap (1, AFC); same locality and habitat data but 8–23.V.2012, 14–29.VIII.2012, C. Alderson & V. Webster // Lindgren funnel traps in canopy of in *Tilia americana* (1, AFC; 1, RWC). **Gloucester Co.**, Bathurst, Daly Point Nature Preserve, 47.6392°N, 65.6098°W, 15–25.VI..2015, C. Alderson & V. Webster // Mixed forest, black Lindgren funnel trap 1 m high (1, AFC). **Kent Co.**, Kouchibouguac National Park, 46.8072°N, 64.9100°W, 21–27.V.2015, 12–24.VI.2015, C. Alderson & V. Webster // Jackpine forest, Lindgren funnel traps, 1 m high (2, AFC; 1, RWC). **Northumberland Co.**, ca. 1.5 km NW of Sevogle, 47.0939°N, 65.8387°W, 14–28.V.2013, C. Alderson & V. Webster // *Populus tremuloides* stand with a few conifers, Lindgren funnel trap 1 m high under *Populus tremuloides* (1, AFC); ca. 2.5 km W of Sevogle, 47.0876°N, 65.8613°W, 1–14.V.2013, 11–25.VI.2014, C. Alderson & V. Webster // Old *Pinus banksiana* stand, Lindgren funnel traps (2, AFC); Upper Graham Plains, 47.1001°N, 66.8154°W, 28.V-10.VI.2014, 24.VI-9.VII.2014, C. Alderson & V. Webster // Old black spruce forest, Lindgren funnel traps (2, AFC). **Queens Co.**, Cranberry Lake P.N.A., 46.1125°N, 65.6075°W, 12–21.V.2009, 21–27.V.2009, 6–14.VIII.2009, R.P. Webster & M.-A. Giguère, coll. // Old red oak forest, Lindgren funnel traps (1, AFC; 2, RWC); C.F.B. Gagetown, 45.7516°N, 66.1866°W, 9–22.V.2013, C. Alderson & V. Webster // Old mixed forest with *Quercus rubra*, Lindgren funnel trap in canopy of *Quercus rubra* (1, AFC). **Restigouche Co.**, Dionne Brook P.N.A., 47.9064°N, 68.3441°W, 31.V–15.VI.2011, 15–27.VI.2011, M. Roy & V. Webster // Old-growth white spruce & balsam fir forest, Lindgren funnel traps (1, CELC; 2, RWC); ca. 3 km SE of Simpsons Field, 47.5277°N, 66.5142°W, 28.V–15.VI.2015, 25.VI–10.VII.2015, C. Alderson & V. Webster // Old cedar & spruce forest with *Populus balsamifera* & *Populus tremuloides*, Lindgren funnel traps (2, AFC). **Sunbury Co.**, Acadia Research Forest, 45.9866°N, 66.3441°W, 13–19.V.2009, 19–25.V.2009, 2–9.VI.2009, 8–13.VII.2009, R.P. Webster & M.-A. Giguère // Red spruce forest with red maple & balsam fir, Lindgren funnel traps (3, AFC; 2 RWC); Gilbert Island, 45.8770°N, 66.2954°W, 25.VII-8.VIII.2012, 13–23.V.2013, C. Alderson, C. Hughes, & V. Webster // Hardwood forest, Lindgren funnel trap in canopy of *Tilia americana* (1) and *Ulmus americana* (1) (2, AFC); Sunpoke Lake, 45.7656°N, 66.5550°W, 15–27.VIII.2012, C. Alderson & V. Webster // Red oak forest near seasonally flooded marsh, Lindgren funnel trap 1 m high under *Quercus rubra* (1, AFC). **York Co.**, 15 km W of Tracy off Rt. 645, 45.6848°N, 66.8821°W, 8–15.VI.2009, 28.VI–7.VII.2009, R.P. Webster & M.-A. Giguère // Old red pine forest, Lindgren funnel traps (2, RWC); 14 km WSW of Tracy S of Rt. 645, 45.6741°N, 66.8661°W, 26.V-2.VI.2010, R. Webster & C. MacKay, coll. // Old mixed forest with red & white spruce, red & white pine, balsam fir, eastern white cedar, red maple & *Populus* sp., Lindgren funnel trap (1, RWC); Fredericton, Odell Park, 45.9539°N, 66.6666°W, 3–15.V.2013, C. Alderson & V. Webster // Hardwood stand, Lindgren funnel trap 1 m high under trees (1, AFC); Charters Settlement, 45.8395°N, 66.7391°W, 9.VII.2008, R.P. Webster // Mixed forest, m. v. light (1, CELC); Douglas, Currie Mountain, 45.9832°N, 66.7564°W, 3–15.V.2013, C. Alderson & V. Webster // Old *Pinus strobus* stand, Lindgren funnel trap in canopy of *Pinus strobus* (1, AFC); Douglas, Currie Mountain, 45.9844°N, 66.7592°W, 3–15.V.2013, 27.V–10.VI.2013, C. Alderson & V. Webster // Mixed forest with *Quercus rubra*, Lindgren funnel trap in canopy of *Quercus rubra* (1), 1 m high under *Quercus rubra* (1) (1, AFC, 1, NBM); Keswick Ridge, 45.9962°N, 66.8781°W, 4–19.VI.2014, C. Alderson & V. Webster // Mixed forest, Lindgren funnel trap 1 m high under trees (1, AFC); Eel River P.N.A., 45.8966°N, 67.6345°W, 21.V–2.VII.2014, 28.VII–12.VIII.2014, C. Alderson & V. Webster // Old-growth eastern white cedar swamp/fen, Lindgren funnel traps (1, AFC; 1, NBM).

####### Distribution in Canada and Alaska.

AK, NT, BC, AB, MB, ON, QC, **NB**, NS, NF (Bousquet et al. 2013). All but one of the new records of *Orthocis punctatus* (Mellié) from NB were based on specimens captured in Lindgren funnel traps. *Orthocis punctatus* is widespread (21 localities) and one of the most common species of Ciidae in NB. Specimens were captured in hardwood, mixed, and conifer forests.

####### Taxonomic notes.

There is evidence that *Orthocis punctatus* comprises at least two species in North America and that they may be conspecific to European species. This problem was first noted by Lawrence (1971), but he kept all known forms under the same name due to the lack of a comparative study of male genitalia. The matter is beyond the scope of this project, and we prefer to attribute the name *Orthocis punctatus* to the species from NB.

###### Plesiocis cribrum

Taxon classificationAnimaliaColeopteraCiidae

Casey, 1898 new to New Brunswick

Fig. 15

####### Material examined.

**New Brunswick, Carleton Co.**, Jackson Falls, “Bell Forest”, 46.2200°N, 67.7231°W, 31.VII-14.VIII.2012, C. Alderson & V. Webster // Rich Appalachian hardwood forest with some conifers, Lindgren funnel trap in canopy of *Fraxinus americana* (1, RWC). **Northumberland Co.**, ca. 1.5 km NW of Sevogle, 47.0939°N, 65.8387°W, 26.VI–8.VII.2013, C. Alderson & V. Webster // *Populus tremuloides* stand with a few conifers, Lindgren funnel trap 1 m high under *Populus tremuloides* (1, RWC); ca. 2.5 km W of Sevogle, 47.0876°N, 65.8613°W, 11–26.VI.2013, C. Alderson & V. Webster // Old *Pinus banksiana* stand, Lindgren funnel trap (1, RWC). **Queens Co.**, Cranberry Lake P.N.A., 46.1125°N, 65.6075°W, 7–22.VI.2011, M. Roy & V. Webster // Old red oak forest, Lindgren funnel trap (1, AFC); C.F.B. Gagetown, 45.7516°N, 66.1866°W, 12–28.VIII.2013, C. Alderson & V. Webster // Old mixed forest with *Quercus rubra*, Lindgren funnel trap in canopy of *Quercus rubra* (1, RWC). **York Co.**, Fredericton, Odell Park, 45.9539°N, 66.6666°W, 10–24.VI.2013, 24.VI–9.VII.2013, C. Alderson & V. Webster // Hardwood stand, Lindgren funnel traps in canopy (3, RWC).

####### Distribution in Canada and Alaska.

BC, AB, MB, QC, **NB** (Bousquet et al. 2013). All new records of *Plesiocis cribrum* Casey from NB were based on specimens captured in Lindgren funnel traps. This species is currently known from six localities in NB and appears to be uncommon. Specimens were captured in hardwood, mixed, and conifer forests. These are the first records of this species from the Maritime Provinces.

##### Tribe Orophiini C.G. Thomson, 1863

###### Octotemnus glabriculus

Taxon classificationAnimaliaColeopteraCiidae

(Gyllenhal, 1827)*

Fig. 16

Cis glabriculus Gyllenhal, 1827: 629
Octotemnus denudatus Casey, 1898: 91, **new synonym**; Dury 1917: 27 (as syn. of Octotemnus laevis Casey)
Octotemnus laevis Casey, 1898: 91, **new synonym**

####### Material examined.

**New Brunswick, Carleton Co.**, Jackson Falls, “Bell Forest”, 46.2204°N, 67.7274°W, 8.VIII.2006, R.P. Webster // Hardwood forest, on polypore fungus on dead standing beech (1, AFC; 1, RWC); same locality but 46.2200°N, 67.7231°W, 6.V.2007, R.P. Webster // Rich Appalachian hardwood forest, on fleshy polypore (bracket) fungi on dead standing beech (1, AFC); same locality and forest type but 12.IX.2008, R.P. Webster // in fleshy polypore mushroom on beech log (1, RWC); same locality and habitat data but 12–19.VI.2008, R. P. Webster // Lindgren funnel trap (1, AFC). **Queens Co.**, Cranberry Lake P.N.A., 46.1125°N, 65.6075°W, 11–18.VI.2009, R.P. Webster & M.-A. Giguère, coll. // Old red oak forest, Lindgren funnel trap (1, RWC). **Sunbury Co.**, Acadia Research Forest, 45.9866°N, 66.3441°W, 9–16.VI.2009, R.P. Webster & M.-A. Giguère // Red spruce forest with red maple & balsam fir, Lindgren funnel trap (1, RWC). **York Co.**, Charters Settlement, 45.8286°N, 66.7365°W, 15.IX.2006, R.P. Webster // Mixed mature forest, on polypore fungi on tree trunk (1, RWC); same locality but 5.V.2005 // Mixed forest, in fleshy polypore fungi on stump (1, CELC); 15 km W of Tracy off Rt. 645, 45.6848°N, 66.8821°W, 1–8.VI.2009, R. Webster & M.-A. Giguère, coll. // Old red pine forest, Lindgren funnel traps (1, AFC; 1, CELC; 3, RWC); Douglas, Currie Mountain, 45.9844°N, 66.7592°W, 24.VI-9.VII.2013, C. Alderson & V. Webster // Mixed forest with *Quercus rubra*, Lindgren funnel trap in canopy of *Quercus rubra* (1, RWC); Canterbury, Eel River P.N.A., 45.8966°N, 67.6345°W, 2–20.VI.2014, C. Alderson & V. Webster // Old-growth eastern white cedar swamp & fen, Lindgren funnel traps (1, NBM; 1, RWC).

####### Distribution in Canada and Alaska.

AK, BC, AB, SK, MB, ON, QC, NB, NS, NF (Bousquet et al. 2013). Most records of *Octotemnus glabriculus* from NB were based on specimens captured in Lindgren funnel traps. This species is currently known from seven localities from hardwood, mixed, and conifer forests in southern NB. Adults were collected from polypore fungi at several sites. This species was previously reported from NB by McNamara (1991) but without supporting data.

####### Taxonomic notes.

The possible synonymy of *Octotemnus glabriculus* (Gyllenhal) and *Octotemnus laevis* (Casey) was first proposed by Lawrence (1971) and corroborated by subsequent molecular analyses (Buder et al. 2008; Lopes-Andrade and Grebennikov 2015). The type locality of *Octotemnus glabriculus* is Sweden, and specimens from England, Germany, Poland, Sweden, and a few other European countries were examined. The type locality of *Octotemnus laevis* is Rhode Island (USA), a locality on the northeastern coast and about 500 linear km south of NB, and specimens from western and eastern localities in Canada and USA were examined. It is important to note that specimens from the same populations with published molecular data of both *Octotemnus glabriculus* and *Octotemnus laevis* (see Buder et al. 2008) were also dissected and compared. The aedeagus in males from USA and Canada are exactly the same as in European specimens. Based on these observations and on previous morphological (e.g., Lawrence 1971) and molecular studies (Buder et al. 2008, Lopes-Andrade and Grebennikov 2015), we propose the synonymization of *Octotemnus glabriculus* and *Octotemnus laevis*. *Octotemnus denudatus* Casey was previously synonymized with *Octotemnus laevis*; we agree with this synonym and, consequently, *Octotemnus denudatus* is here proposed as a new synonym of *Octotemnus glabriculus*. *Octotemnus* Mellié is highly diversified in the Palaearctic region (Kawanabe 2002), and only *Octotemnus glabriculus* is officially reported from North America. There seems to be no native species of the genus restricted to North America. *Octotemnus glabriculus* is widespread in the Holarctic region and seems to be closely related to *Octotemnus omogensis* Miyatake from Japan and *Octotemnus rugosopunctatus* Drogvalenko from the Caucasus.

## Supplementary Material

XML Treatment for Ceracis singularis

XML Treatment for Ceracis thoracicornis

XML Treatment for Cis americanus

XML Treatment for Cis angustus

XML Treatment for Cis creberrimus

XML Treatment for Cis fuscipes

XML Treatment for Cis horridulus

XML Treatment for Cis levettei

XML Treatment for Cis striatulus

XML Treatment for Cis submicans

XML Treatment for Dolichocis laricinus

XML Treatment for Dolichocis manitoba

XML Treatment for Hadreule elongatula

XML Treatment for Malacocis brevicollis

XML Treatment for Orthocis punctatus

XML Treatment for Plesiocis cribrum

XML Treatment for Octotemnus glabriculus
